# Supplementary material for: Reconciling Mining with the Conservation of Cave Biodiversity: A Quantitative Baseline to Help Establish Conservation Priorities
Source: PLoS One. 2016 Dec 20;11(12):e0168348. doi: 10.1371/journal.pone.0168348 (PMC5173368; doi:10.1371/journal.pone.0168348)
Supplement: S1 Dataset — (ZIP) [file pone.0168348.s002.zip › Taxa/Serra Norte/SN_2013/PES/Lista PESE-01.pdf]

CAVIDADE PESE-0001

Classe	Ordem	Fam/Outros	Gên/Outros	Espécie	S-U	U-S	Zon.
Annelida	Oligochaeta			sp.	2		P
Arachnida	Acari	(Oribatida)		sp.1		X	E
Arachnida	Acari	(Parasitengonina)		sp.3		X	E
Arachnida	Acari	Anystidae	*Erythracarus*	*nasutus*		X	E
Arachnida	Acari	Rhagidiidae		sp.1	X		P
Arachnida	Acari	Trombiculidae		sp.1		X	P
Arachnida	Amblypygi	Phrynidae	*Heterophrynus*	*longicornis*	2	1	E/P
Arachnida	Araneae	Araneidae	*Alpaida*	*antonio*	X	X	P
Arachnida	Araneae	Ctenidae		jovem	3		E
Arachnida	Araneae	Pholcidae	*Mesabolivar*	*paraensis*	X	X	P
Arachnida	Araneae	Segestriidae	*Ariadna*	sp.2	X		P
Arachnida	Araneae	Theridiosomatidae	*Plato*	sp.1	X		P
Arachnida	Opiliones	Sclerosomatidae	*Prionostema*	sp.1		X	E
Arachnida	Opiliones	Stygnidae	*Protimesius*	*gracilis*	1		E
Arachnida	Palpigradi	Eukoeneniidae	*Eukoenenia*	sp.1	X		P
Arachnida	Pseudoscorpiones	Chernetidae	*Spelaeochernes*	sp.1	X	X	P
Arachnida	Pseudoscorpiones	Chthoniidae	*Pseudochthonius*	sp.1		X	E
Diplopoda	Polydesmida	Fuhrmannodesmidae		sp.1		X	E
Diplopoda	Polydesmida	Fuhrmannodesmidae		sp.2		X	E
Diplopoda	Polydesmida	Fuhrmannodesmidae		sp.3		X	P
Entognatha	Collembola	Paronellidae		sp.1	X	X	E/P
Entognatha	Diplura	Campodeidae		sp.1		X	E
Insecta	Coleoptera	Elateridae		jovem		X	P
Insecta	Coleoptera	Tenebrionidae		jovem	X		P
Insecta	Diptera	Cecidomyiidae	Cecidomyiinae	sp.	X	X	E
Insecta	Diptera	Psychodidae	*Sciopemyia*	*sordellii*	X	X	E/P
Insecta	Diptera	Tipulidae	Tipulinae	sp.	X		P
Insecta	Heteroptera	Reduviidae	Emesinae	jovem		1	P
Insecta	Hymenoptera	Formicidae	*Camponotus*	sp.1		X	E
Insecta	Hymenoptera	Formicidae	*Nylanderia*	sp.1	X		E
Insecta	Hymenoptera	Formicidae	*Nylanderia*	sp.3		X	E
Insecta	Hymenoptera	Formicidae	*Pachycondyla*	*striata*	X		E
Insecta	Hymenoptera	Formicidae	*Pheidole*	sp.2	X	X	E/P
Insecta	Isoptera	Termitidae		sp.		X	P
Insecta	Lepidoptera	Noctuoidea		sp.5		1	E
Insecta	Lepidoptera			jovem	X		P
Insecta	Orthoptera	Phalangopsidae	*Paraclodes*	sp.	3		E
Insecta	Orthoptera	Phalangopsidae	*Phalangopsis*	sp.	1	10	P
Insecta	Thysanura	Nicoletiidae		sp.1	X	X	P
Gastropoda	Pulmonata	Systrophiidae	*Happia*	sp.		X	P
Malacostraca	Isopoda	Platyarthridae		sp.2	X	X	P
Amphibia	Anura	Strabomantidae	*Pristimantis*	cf. *fenestratus*	1		P
Mammalia	Chiroptera	Emballonuridae	*Peropteryx*	sp.	1		P
